# Microstructure and ionic conductivity investigation of samarium doped ceria (Sm_0.2_Ce_0.8_O_1.9_) electrolytes prepared by the templating methods

**DOI:** 10.55730/1300-0527.3379

**Published:** 2022-02-25

**Authors:** Dilara GÜÇTAŞ, Vedat SARIBOĞA, M.A. Faruk ÖKSÜZÖMER

**Affiliations:** Department of Chemical Engineering, Faculty of Engineering, İstanbul University-Cerrahpaşa, İstanbul, Turkey

**Keywords:** SOFC, electrolyte, SDC20, sintering, grain growth, templating

## Abstract

Sm_0.2_Ce_0.8_O_1.9_ (SDC20) electrolytes were synthesized with cellulose templating (CT) and PVA templating (PVAT) methods. Powder characteristics were examined using TG/DTA, XRD, and SEM. Pellets are sintered at various temperatures for different durations. Mean grain sizes were calculated from SEM micrographs using the linear intercept method. Grain growth behavior of the electrolytes was investigated and the dominant diffusion mechanism was examined. The grain growth activation energies were obtained for the first time for the mentioned electrolytes prepared by the mentioned methods. The ionic conductivities were calculated by electrochemical impedance spectroscopy. The highest ionic conductivity value was found to be 0.050 S cm^−1^ for the cellulose templating method.

## 1. Introduction

Solid oxide fuel cells (SOFCs) are recognized as the most effective energy conversion devices with future applications. Lowering the operating temperature of SOFCs is extremely important for efficient operation [1]. This can be achieved by using the effective electrolyte under the right conditions. The ionic conductivity of the electrolyte is the most crucial aspect affecting the efficiency of solid oxide fuel cells. The electrolyte is responsible for ion transport within the cell to create electrochemical reactions. Numerous chemical materials are reported in the literature as the best electrolyte for SOFCs, but among all the doped ceria compounds show outstanding performance due to their specific properties, such as low activation energy in the 500–800 °C temperature range [2]. The ionic conductivity is highly affected by the concentration and the ionic radius of the dopant cations. Among the various dopants used, Sm^3+^, and Gd^3+^ are favorable for increasing the ionic conductivity [3].

There are many methods reported to prepare doped ceria compounds such as coprecipitation methods by using precipitation agents like ammonium hydroxide [4], oxalic acid [5], and ammonium carbonate [6], or auto-combustion methods using fuels such as citric acid [7], glycine [8], ethylene glycol [9], and EDTA [10]. Additionally, templating methods provide extremely pure and fine particle-sized ceramic powders. In this method, metal cations are prepared in solution and trapped in a template. The template burns out with calcination and, the resulting combustion heat contributes to the crystallization of the obtained ceramic. Compounds such as chitosan, polyvinyl alcohol, and cellulose can be used as templates for the production of SDC20 electrolytes. [11–13].

Another crucial parameter to achieve high-performance electrolytes is considered the sintering process. Sintering at high temperatures is essential to achieve a dense, nonporous structure. Knowing the effective sintering temperature and time is extremely important in terms of efficiency, time, and cost.

The ionic conductivity is affected by the electrolyte’s grain size after sintering. In general, the grain size of ceramics increases as the sintering temperature increases. The grain growth of the electrolyte is usually controlled by grain boundary diffusion and lattice diffusion. The dominant mechanism during sintering significantly affects the properties of the final product.

Studies on grain growth kinetics of SDC20 are quite limited in the literature. The most detailed study was carried out by our group using the chitosan templating method. In our work, the dominant grain growth mechanism during sintering was determined by sintering SDC20 electrolytes at different temperatures [14].

In this work Sm_0.2_Ce_0.8_O_1.9_ (SDC20) electrolytes were synthesized by the cellulose templating and polyvinyl alcohol (PVA) templating methods. Samples were sintered at 1200, 1300, and 1400 °C for 5 min, 1 h, and 6 h each. The grain sizes of the electrolytes were determined and the relationship between the synthesis method and sintering kinetics was investigated for the first time to the best of our knowledge.

## 2. Materials and methods

SDC20 (Sm_0.2_Ce_0.8_O_1.9_, SDC20) electrolytes were synthesized according to the CT and PVAT methods reported in the literature [15,16].

### 2.1. Cellulose templating method

**Figure 1:**
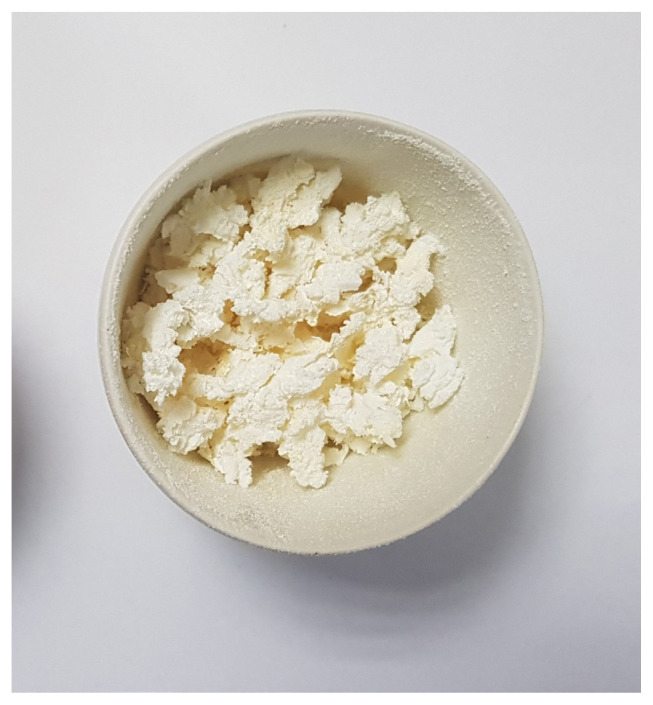
The foamy SDC20 ceramic structure after calcination.

Ce(NO_3_)_3_.6H_2_O (Alfa Aesar, 99.5%), Sm(NO_3_)_3_.6H_2_O (Alfa Aesar, 99.9%) were dissolved in water and 1 M solution was prepared. Then, the solution was dropped onto 125 mm diameter ashless cellulose filter paper (Macharey-Nagel, MN 640 de, ≤2 μm) in the ratio of 1 mL solution per filter paper. Afterward, the penetrated cellulose papers were burned in a preheated oven (500 °C) for 30 min and then calcined at 600 °C for 6 h. The remaining foamy residue (Figure 1) consists of pure metal oxides. Then, the residue ground in an agate mortar and pure SDC20 powders were obtained.

### 2.2. PVA templating method

**Figure 2:**
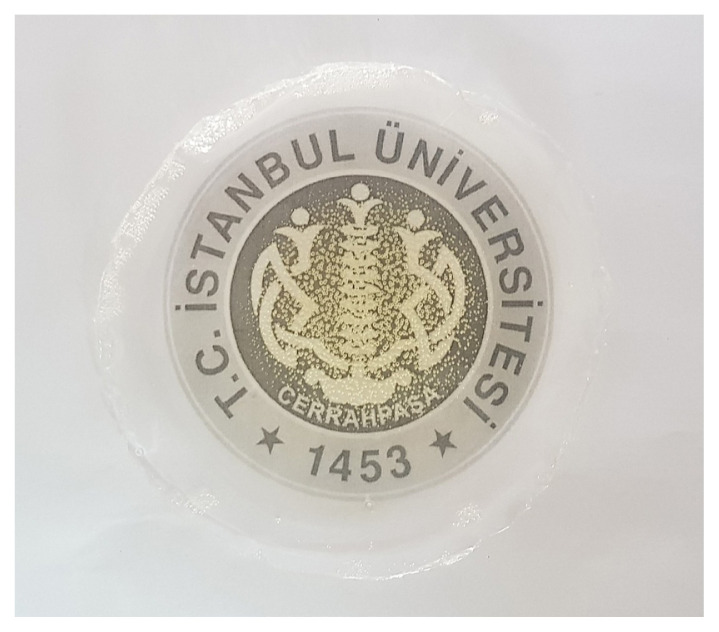
Clear and flexible PVA-cation film.

A total of 0.5 M cation solution was prepared by dissolving Ce(NO_3_)_3_.6H_2_O (Alfa Aesar, 99.5%) and Sm(NO_3_)_3_.6H_2_O (Alfa Aesar, 99.9%) in distilled water in the correct stoichiometric amounts. Subsequently, an aqueous PVA (5 wt.%) solution was prepared and added to the cation solution with the molar ratio of PVA to cations P: C = 1. Then, a homogeneous solution was obtained by mixing the solution for 48 h. Afterward, the solution was poured into a flat surface and left to dry at room temperature for 4 days, and a clear and flexible film (Figure 2) was obtained. The calcination of the precursor was carried out at 600 °C for 6 h and the resulting foamy material was ground in an agate mortar.

The ceramic powders were pressed into 10 mm diameter pellets via stainless steel pellet die. To increase their strength, cold isostatic press was applied. Later, pellets were sintered at 1200–1400 °C temperature range for 5 min, 1 h, and 6 h with a 5 °C/min heating rate.

The thermogravimetric analysis was applied to the precursors prior to calcination with Seiko SII Exstar 6000 thermogravimetric/differential thermal analysis between 30 °C and 800 °C.

XRD analysis was applied to the samples to explore the crystalline structure. The powders after calcination and the powders of ground pellets were studied by XRD using Rigaku D/max-2200 diffractometer with CuKα radiation in an angular region of 2θ = 20°– 80°.

The theoretical densities were calculated by Equation (1), and the sample densities were measured using the Archimedes method.


(1) $$\rho =\frac{\left[xM_{d}+(1-x)M_{ce}+\left(2-\frac{x}{2}\right)M_{O}\right]}{N_{A}a^{3}}$$

where ρ is the theoretical density (g cm^−3^),

x is the dopant concentration,
M_d_ is the molecular weight of the dopant,
M_Ce_ is the molecular weight of the cerium,
M_O_ is the molecular weight of the oxygen,
a is the lattice parameter, and
N_A_ is the Avogadro number.

**Figure 3:**
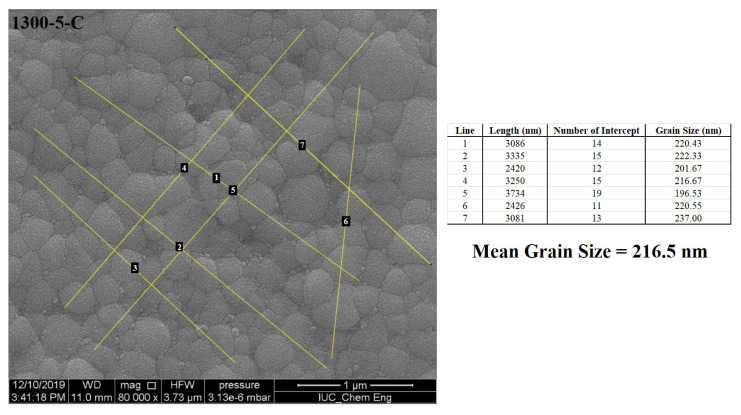
The linear intercept method for the mean grain size calculation.

Microstructural properties of the sintered electrolytes were investigated via scanning electron microscope (SEM-EDX, FEI Quanta FEG-450). The average grain size of the electrolytes was calculated using the SEM micrographs, via the linear intercept method from three different micrographs. Then, a line through the micrograph was drawn, and the number of grain boundaries intersecting the line was counted. By dividing the number of intersections by the real line length, the mean grain size of the electrolytes was calculated. The estimation of the mean grain size of the electrolytes using only one micrograph is given in Figure 3. This process is repeated 3 times for all samples.

Electrical properties of the electrolytes were measured by using a GAMRY Instruments PCI4G750/46070 Potentiostat in the 250–800 °C temperature range. The Nyquist curves were plotted and, the sample resistances (R) were obtained. Then, the total ionic conductivities of the samples were calculated using the equation below.


(2) $$\sigma_{total}=\frac{l}{A.R_{total}}$$

where σ_total_ is the total ionic conductivity,

A is the crosssectional area of the sample
l is the sample thickness, and
R_total_ is the total resistance.

## 3. Results

### 3.1. Powder characterization

**Figure 4:**
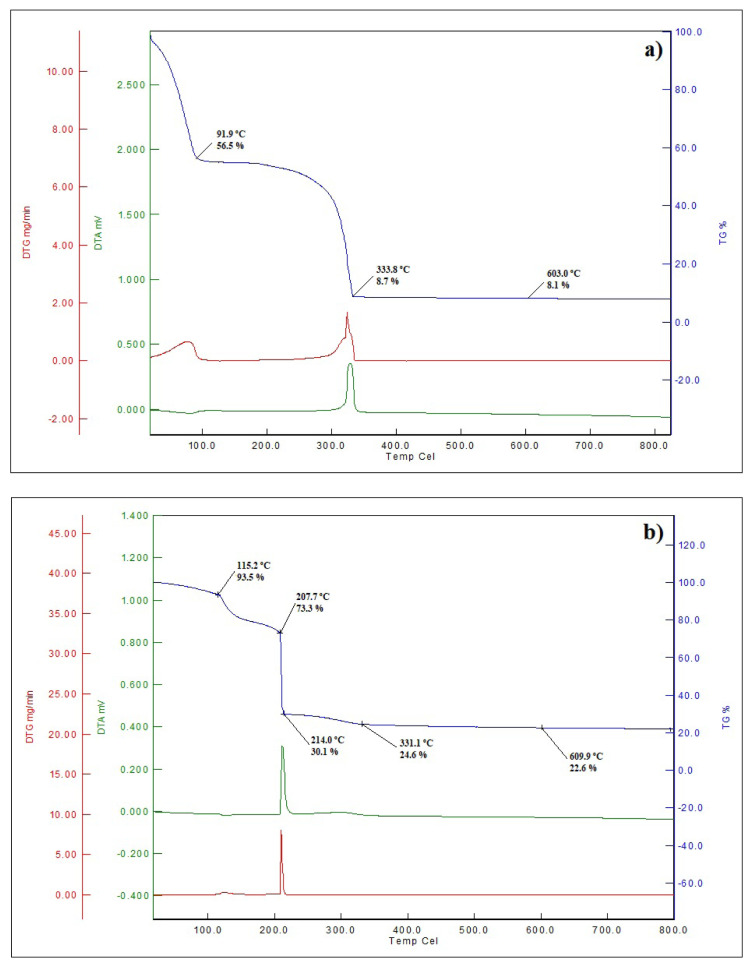
TG/DTA graph of the powders; (a) prepared by CT method, (b) prepared by PVAT method.

The thermal behavior of the both precursors prior to calcination were investigated with TG/DTA and the charts are given in Figures 4a and 4b.

Figure 4a shows the TG/DTA results of the cation impregnated filter paper. The first weight loss (about 40%) resulted from the evaporation of the water. Then, a weight loss of 47% occurred at 333 °C and, SDC20 was obtained. Although the decomposition of the filter paper itself occurs at around 500 °C, the presence of the cations lowered the decomposition temperature and made it possible to form pure SDC20 in a single step . A small mass loss of 0.6% was observed until 600 °C and, the remaining mass can assume to be SDC20.

Figure 4b shows the TG/DTA results of the PVA-cation film. The initial weight loss of 6.5% may be attributed to the evaporation of the moisture in the film. Then, at 115 °C the decomposition of the PVA started. A sudden weight loss of 43% occurred and, SDC20 formed in the temperature range of 207–214 °C. Normally, the decomposition of pure PVA starts at 240 °C [17], but the presence of the cations lowered the decomposition temperature. The combustion of organic residues continued up to 330 °C and, about 2% of weight loss was observed until 600 °C. No remarkable weight loss was detected after this temperature and both powders were calcined at this temperature.

**Figure 5:**
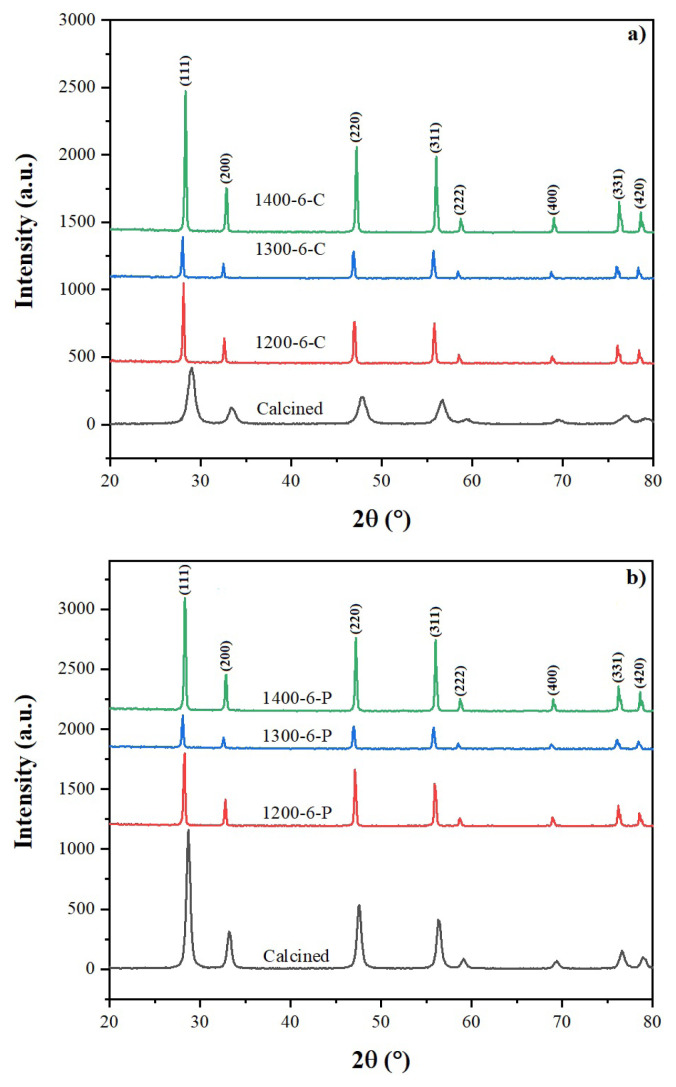
XRD patterns of the electrolytes after calcination and sintering a) prepared by CT method, b) prepared by PVAT method.

XRD patterns of the samples calcined at 600 °C and sintered at 1200, 1300, and 1400 °C for 6 h are given in Figure 5. XRD results show that crystallization of the powders occurred after calcination at 600 °C. The samples consist of single-phase CeO_2_ with a cubic fluorite structure (JCPDS Card No: 34-394). No peak of samarium oxide was observed which shows the dopant cation was entirely placed in the lattice. The electrolytes are named to state the synthesis route and, sintering condition applied. For example, 1200-6-C refers to the sample synthesized by the CT method and sintered at 1200 °C for 6 h.

**Figure 6:**
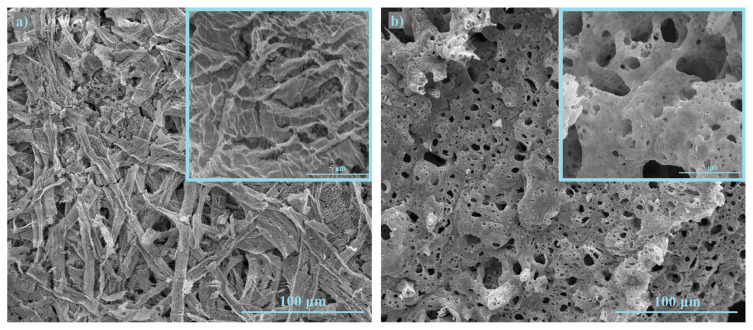
SEM micrographs of the powders; (a) prepared by CT method, (b) prepared by PVAT method.

CT method includes penetration of cation solution inside the pores of the cellulose paper. The cellulose paper has a tightly packed structure with a low void volume. When the cerium-samarium solution has penetrated the pores and burned at 500 °C, the mixture of decomposed compounds created and was trapped in the cellulose paper [11]. Figure 6a shows SEM image of SDC20 powders after the filter paper is burned out. After burning the filter paper there is a networked fiber structure of Sm-Ce-O mixture, which consists of many mesh-like regions. On the other hand, the PVAT method includes the dopants incorporated into the ceria lattice via combustion for pure phase evolution [16]. Figure 6b shows SEM image of SDC20 powders after the calcination of the PVA-cation film and, the structure consists of sponge-like regions due to the release of large amounts of gases during calcination.

### 3.2. Sintering and microstructure

**Figure 7:**
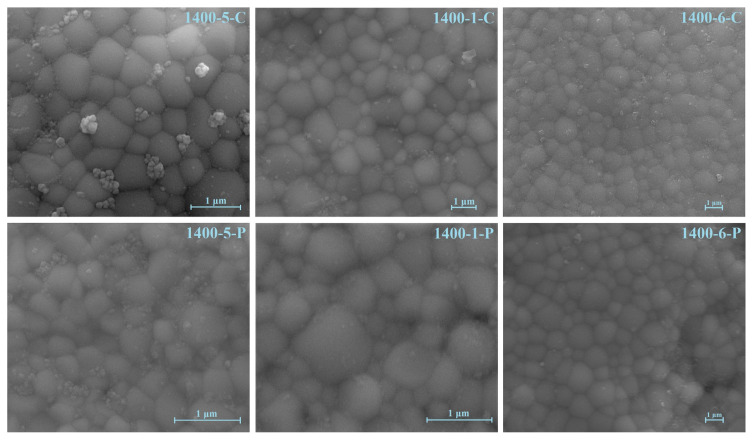
SEM micrographs of the pellets.

Figure 7 shows SEM micrographs of the electrolytes sintered at 1400 °C for 5 min, 1 h, and 6 h.

The samples prepared by both methods reached a dense and nonporous structure at 1400 °C, independent of the sintering time. However, it could be seen that the samples prepared with the cellulose templating method are denser than the ones prepared with PVA templating method. At 1400 °C, the grains can be clearly distinguished and reached a hexagonal structure, regardless of the sintering time. Increasing the sintering time allowed the grains to grow noticeably. Longer sintering times are required to ensure homogeneous grain size distribution.

Density measurements of the sintered pellets were measured via the Archimedes method and all density values are given in Table 1.

For both methods, an increase in the sintering temperature gives higher density values. A relative density value greater than 88% was achieved under all sintering conditions. The samples prepared by the CT method have greater measured and relative density values than the samples prepared by PVAT method. Also, the densest material was the 1400-6-C sample, which was produced by the CT method and sintered at 1400 °C for 6 h. Although the highest density is achieved by sintering at 1400 °C for 6 h, it is possible to achieve comparable densities by sintering at 1300 °C. This means great advantage in terms of time and energy consumption for SDC20 materials.

Detailed grain growth analysis for polycrystalline structures is difficult due to the change of grain boundary energy which causes a change in the grain growth kinetic from boundary to boundary. To simplify the grain growth kinetic, grain boundary energy is presumed to be constant, and the grain growth kinetics can be explained with the following equations:


(3) $$D^{n}-D_{0}^{n}=Kt,$$

(4) $$K=K_{0}e^{-\frac{Q}{RT}}$$

where D expresses the average grain size of the electrolyte,

D_0_ is the grain size of the initial polycrystalline powders at the time (t = 0),
Q is the activation energy of grain growth,
K_0_ is the rate constant,
t is the time, and

n is an exponent, which is an integer ranging from 1 to 4 depending on various kinetic parameters involved in the growth process [18].

Sintering occurs through different diffusion mechanisms. The value of n changes according to the dominant diffusion mechanism. If n = 3, then the volume diffusion is dominant during sintering. If n = 4 then, the grain boundary diffusion is dominant [19].

The grain size of the starting powder (D_0_) is quite small opposed to the electrolytes grain size (D), so D_0_ can be neglected and, Equation (3) can be simplified as follows:


(5) $$\frac{D^{n}}{t}=K_{0}e^{-\frac{Q}{RT}}$$

If the same equation written in logarithmic scale:


(6) $$ln\frac{D^{n}}{t}=lnK_{0}-\frac{Q}{RT}$$

The grain growth exponent (n) can be estimated via the slope of the ln(D) – ln(t) plot if the activation energy is considered constant. Then, using Equation (6) it is possible to estimate the activation energy.

The average grain size of the electrolytes was calculated using the linear intercept method and given in Table 2. The highest grain size values for CT and PVAT methods are 1066.8 ± 10.4 nm and 1074.7 ± 8.1 nm, respectively. Both values are achieved after sintering at 1400 °C for 6 h. The lowest grain sizes for both methods at the same order are 122.2 ± 3.7 nm and 145.7 ± 3.0 nm. These values were achieved for 5 min sintering at 1200 °C. Increasing the sintering temperature caused the grain size to increase monotonically. Also, longer sintering times increase the average grain size of the electrolytes, but this increase is not linear with time.

**Figure 8:**
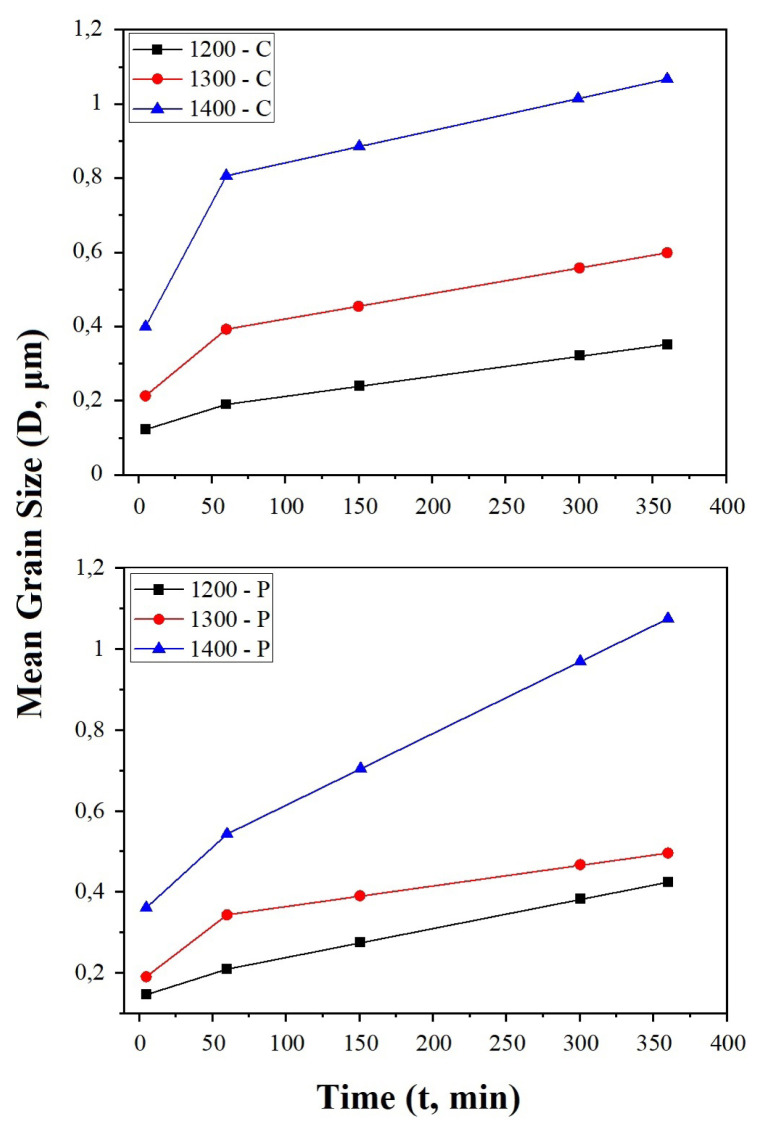
The effect of sintering conditions on the mean grain size.

Figure 8 represents the change of the average grain sizes with sintering conditions. The grain growth at 1200 °C showed an almost linear increase with sintering time. At 1300 °C and 1400 °C, as a result of increasing the sintering time from 5 min to 1 h, an increase of 1.5–2 times in the grain sizes was observed. Similarly, when the sintering time is increased to 6 h, an increase of 1.5 times is observed. This shows the grain growth is limited at a certain point as in our previous work [14].

**Figure 9:**
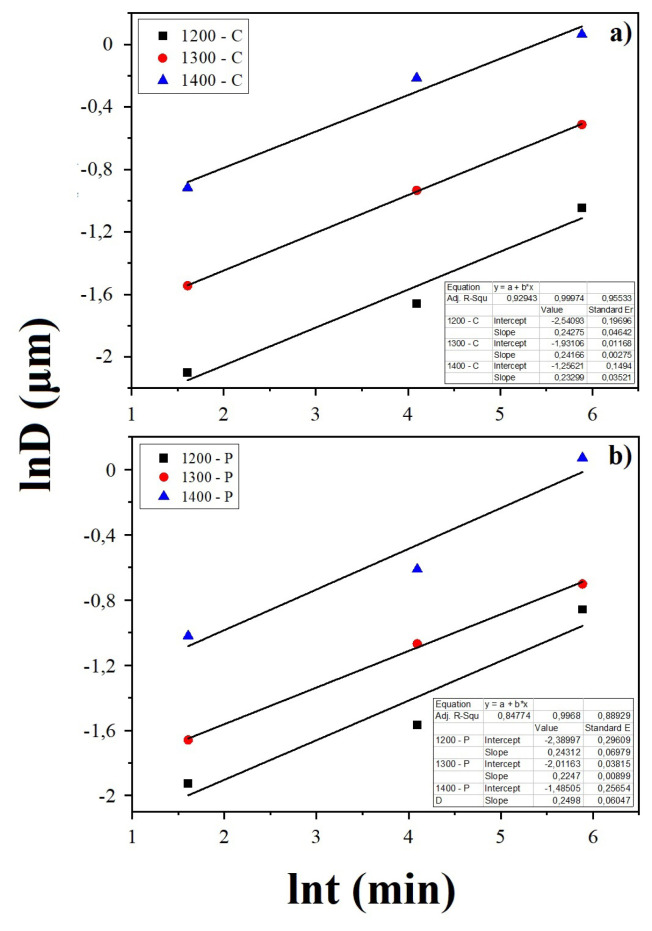
lnD-lnt plot for a) CT method, b) PVAT method.

The grain growth exponent “n” was calculated using Equation (6) and the dominant diffusion mechanism during sintering was determined. Figure 9 shows the lnD – lnt plot of the samples. The slope of this plot gives the value of 1/n. For all samples produced by both methods, the n = 4 was calculated. That shows the grain boundary diffusion is the dominant mechanism during the sintering of the electrolyte. The driving force for sintering comes from the defective structure of the grain boundaries. The lattice diffusion occurs slower than the grain boundary diffusion. Also, the grain boundary diffusion contributes more to the densification of the electrolyte rather than grain growth [20].

**Figure 10:**
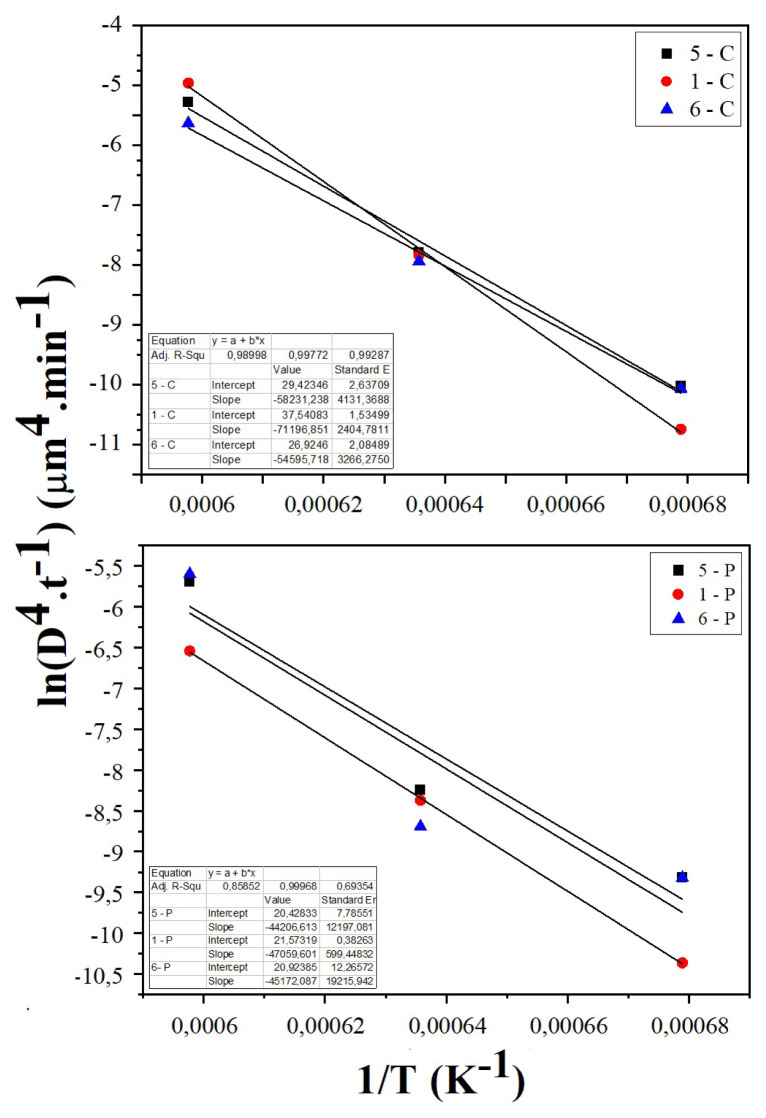
Arrhenius plot for the grain growth.

The sintering activation energies of the samples were calculated using Equation (6). Figure 10 shows Arrhenius plot of lnD^4^/t – 1/T. The calculated activation energies are given in Table 3. For the samples prepared by the CT method, the sintering activation energies are calculated as 484.2, 591.9, and 453.9 kJ mol^−1^ for 5 min, 1 h, and 6 h of sintering, respectively. The sintering activation energies of the samples prepared by the PVAT method are calculated as 367.5, 391.3, and 275.5 kJ mol^−1^ for 5 min, 1 h, and 6 h of sintering, respectively. There is no recorded sintering activation energy for SDC20 synthesized by the CT and the PVAT methods in the literature. However, the activation energy of sintering was found to be 474.4 kJ mol^−1^ for the chitosan templating method [14]. Also, similar values have been noted for other oxide electrolytes such as 669 kJ mol^−1^ for La_0.8_Sr_0.2_Ga_0.83_Mg_0.17_O_2.815_ [21], 697 kJ mol^−1^ for CeO_2_ [22], 580 kJ mol^−1^ for yttria-stabilized zirconia [23].

### 3.3. Conductivity measurements

**Figure 11:**
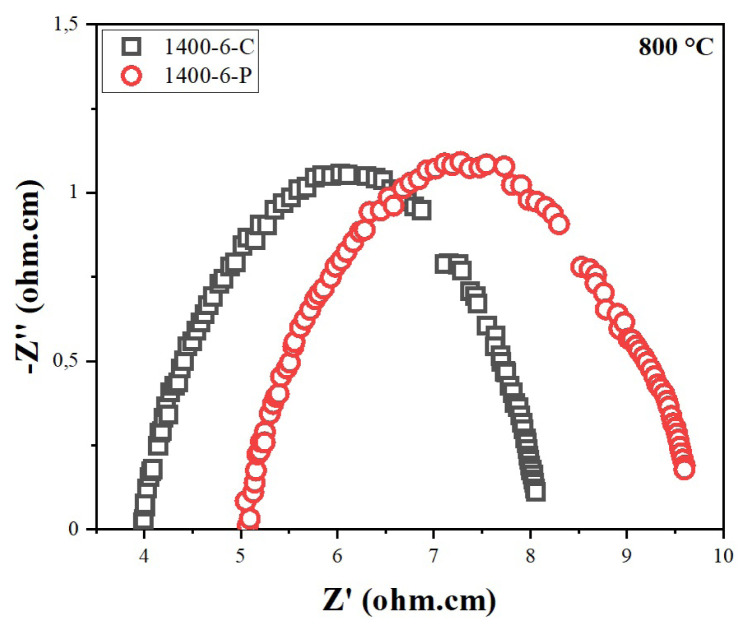
Impedance spectra of the CT and PVAT electrolytes measured at 800 °C.

The electrical properties of the samples were determined by electrochemical impedance spectroscopy (EIS). Impedance measurements were applied to samples sintered at 1400 °C for 6 h. Figure 11 represents the impedance spectra measured at 800 °C for CT and PVAT samples. At this temperature, the total ionic conductivity of the sample prepared by the CT method was much higher (0.050 S cm^−1^) than the sample prepared by the PVAT method (0.037 S cm^−1^). This difference between the two samples also agrees with the SEM and density analysis considering the PVAT method results in a less dense structure. In this case, it was concluded that using cellulose as a templating agent against PVA gives better results.

**Figure 12:**
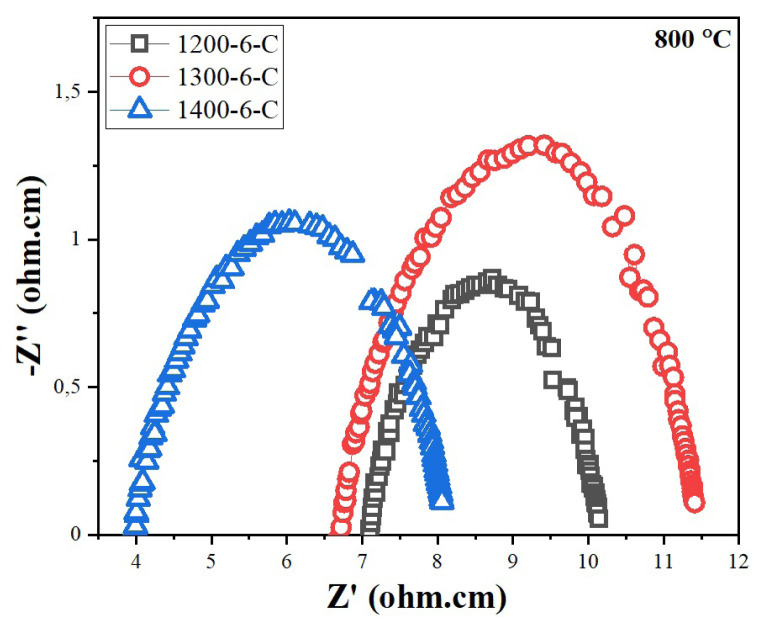
Impedance spectra of the samples prepared by cellulose templating method.

Since the CT method gives better results, EIS analysis also applied the samples sintered at 1200 and 1300 °C, and the impedance spectra are given in Figure 12. The total conductivity values, area specific resistivity (ASR) values, and activation energies are given in Table 4. Specific resistance is the inherent property of a material. It is defined as the resistance offered per unit length and unit crosssectional area of the electrolyte when a known quantity of voltage is applied at its end. The reciprocal of the specific resistance gives the conductivity. Samples sintered at 1200 and 1300 °C have almost the same conductivity values which are 0.030 and 0.033 S cm^−^1, respectively, but at 1400 °C the total conductivity increased significantly.

Jaiswal et al. [24] calculated the ionic conductivity of SDC20 synthesized via auto-combustion method at 1350 °C as 0.0133 S cm^−1^. Spiridigliozzi et al. [25] reported the ionic conductivity as 0.048 S cm^−1^ at 1300 °C for the coprecipitation route. Ma et al. [12] calculated the conductivity as 0.033 S cm^−1^ at 1300 °C for the PVAT method. Özdemir et al. [15] estimated the ionic conductivity of SDC20 as 0.031 S cm^−1^ at 1200 °C for the CT method. This study has proved that both cellulose templating and PVA templating methods are more effective than other methods in the literature for preparing high-performance SDC20 electrolytes.

**Figure 13:**
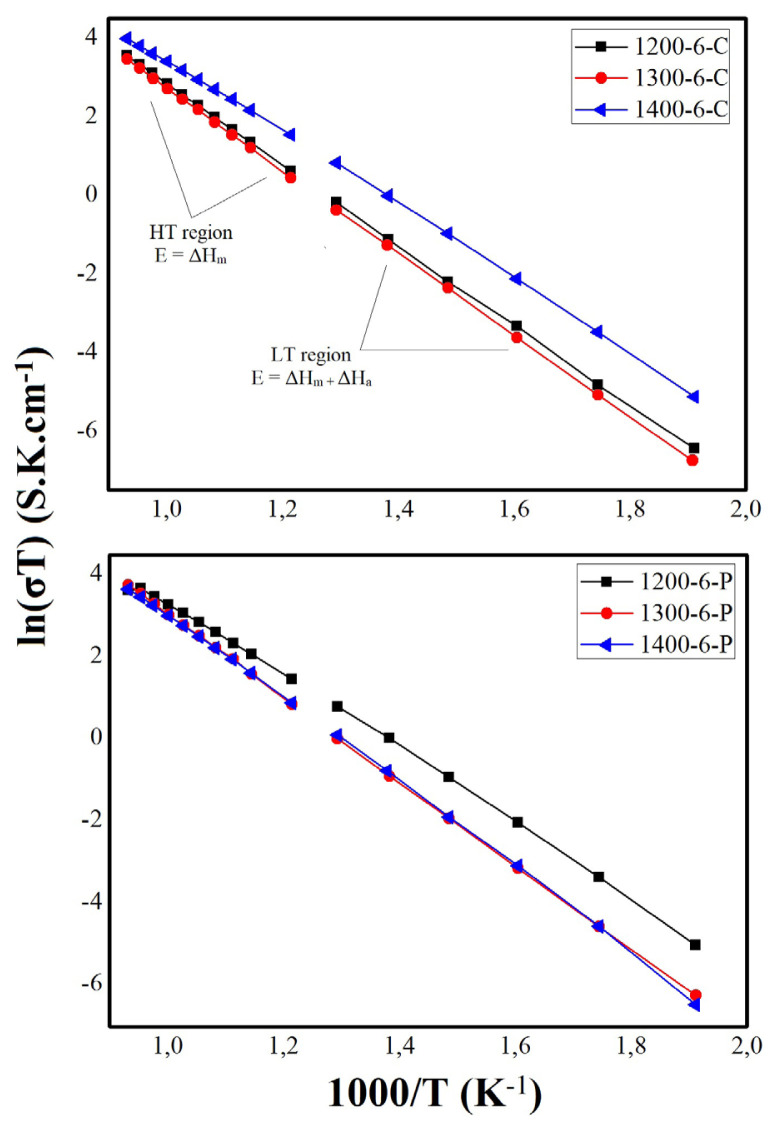
Arrhenius plots of the electrolytes.

Figure 13 shows the temperature dependence of the sample conductivities. The Arrhenius plots appear linear but they consist of two regions named LT (low temperature) and HT (high temperature) according to the change of the linearity of the plot due to the change in the conduction mechanism, which generally starts at 500 °C [26]. The oxygen ionic conductivity of doped ceria materials can be explained as follows:


(7) $$\text{At low temperatures: }\sigma =\frac{\sigma_{0}}{T}e^{\frac{-(\Delta H_{m}+\Delta H_{a})}{kT}},$$

(8) $$\text{At high temperatures: }\sigma =\frac{\sigma_{0}}{T}e^{\frac{-(\Delta H_{m})}{kT}},$$

where ΔH_m_ is the migration enthalpy of the oxygen ions and ΔH_a_ is the association enthalpy of the dopant ion with the oxygen vacancies [27], k is the Boltzmann constant (8.617 × 10^−5^ eVK^−1^), and T is temperature. For the cellulose templating method, calculated HT and LT activation energies are similar at 1200 and 1300 °C, but at 1400 °C, there is a significant decrease at both HT and LT activation energies. Consequently, the high ionic conductivity of the 1400–6-C electrolyte can be explained via decrease in the activation energy.

## 4. Conclusion

SDC20 electrolytes were successfully synthesized by the simple and fast cellulose templating and PVA templating methods and characterized by TGA/DTA, XRD, EIS, and SEM analysis. SDC20 powders with a single fluorite phase were obtained after the calcination at 600 °C. Pellets were prepared with the CIP process and sintered at 1200–1400 °C temperature range for 5 min, 1 h, and 6 h each. A relative density of over 88% was reached in all samples. The grain growth kinetics is examined with SEM images. The average grain sizes were determined via the linear intercept method. The dominant diffusion mechanism was found to be the grain boundary diffusion and, the activation energy of the sintering was calculated. The highest ionic conductivity value (0.050 S cm^−1^) was achieved for the 1400-6-C sample. The cellulose templating method is a more effective method to prepare mixed oxide structures than the precipitation and solid-state methods, as it provides higher performance electrolytes.

## Figures and Tables

**Table 1:** Measured, theoretical, and relative densities.

Sample	Measured density (g cm^−3^)	Theoretical density (g cm^−3^)	Relative density (%)
1200-5-C	6.31	7.14	88.35
1200-1-C	6.49	7.14	90.99
1200-6-C	6.68	7.12	93.75
1300-5-C	6.45	7.12	90.59
1300-1-C	6.66	7.12	93.58
1300-6-C	6.71	7.10	94.38
1400-5-C	6.66	7.12	93.51
1400-1-C	6.81	7.14	95.38
1400-6-C	6.89	7.14	96.58
1200-5-P	6.32	7.14	88.57
1200-1-P	6.42	7.14	89.99
1200-6-P	6.45	7.14	90.33
1300-5-P	6.41	7.12	90.03
1300-1-P	6.47	7.12	90.91
1300-6-P	6.49	7.12	91.17
1400-5-P	6.46	7.12	90.73
1400-1-P	6.57	7.14	92.07
1400-6-P	6.64	7.14	93.02

**Table 2:** Mean grain sizes of all samples.

Sample	Mean grain size (D, nm)		
	5 min	1 h	6 h
1200 - C	122.2 ± 3.7	189.8 ± 4.7	351.5 ± 5.0
1300 - C	213.3 ± 2.9	392.6 ± 7.0	598.9 ± 9.0
1400 - C	399.5 ± 5.0	706.3 ± 10.2	1066.8 ± 10.4
1200 - P	145.7 ± 3.0	208.7 ± 5.3	423.7 ± 5.2
1300 - P	190.3 ± 3.6	343.2 ± 7.0	495.6 ± 5.7
1400 - P	360.4 ± 5.1	542.5 ± 7.9	1074.7 ± 8.1

**Table 3:** Estimated grain growth activation energies.

Sample	Grain growth activation energy (Q, kJ/mol)
5 - C	484.2
1 - C	591.9
6 - C	453.9
5 - P	367.5
1 - P	391.3
6 - P	275.5

**Table 4:** EIS analysis results for the electrolytes prepared by the CT method.

Electrolyte	Total ionic conductivity (S cm^−1^, 800 °C)	Area specific resistivity (cm S^−1^, 800 °C)	E_A_ (high temperature, eV)	E_A_ (low temperature, eV)	ΔE (eV)
1200-6-C	0.030	33.333	0.872	0.892	0.020
1300-6-C	0.033	30.303	0.896	0.916	0.020
1400-6-C	0.050	20.000	0.742	0.829	0.087
